# 2-(4-Fluoro­phen­yl)-1-(phenyl­sulfin­yl)­naphtho­[2,1-*b*]furan

**DOI:** 10.1107/S1600536811034155

**Published:** 2011-08-27

**Authors:** Pil Ja Seo, Hong Dae Choi, Byeng Wha Son, Uk Lee

**Affiliations:** a Department of Chemistry, Dongeui University, San 24 Kaya-dong Busanjin-gu, Busan 614-714, Republic of Korea; b Department of Chemistry, Pukyong National University, 599-1 Daeyeon 3-dong, Nam-gu, Busan 608-737, Republic of Korea

## Abstract

In the title compound, C_24_H_15_FO_2_S, the 4-fluoro­phenyl ring makes a dihedral angle of 19.43 (4)° with the mean plane of the naphtho­furan fragment. The dihedral angle between the phenyl ring and the mean plane of the naphtho­furan fragment is 85.83 (4)°. In the crystal, mol­ecules are linked by weak inter­molecular C—H⋯O hydrogen bonds.

## Related literature

For the pharmacological activity of naphtho­furan compounds, see: Goel & Dixit (2004 ▶); Hagiwara *et al.* (1999 ▶); Piloto *et al.* (2005 ▶). For structural studies of related 2-ar­yl-1-(phenyl­sulfin­yl)naphtho­[2,1-*b*]furan derivatives, see: Choi *et al.* (2009*a* ▶,*b* ▶).
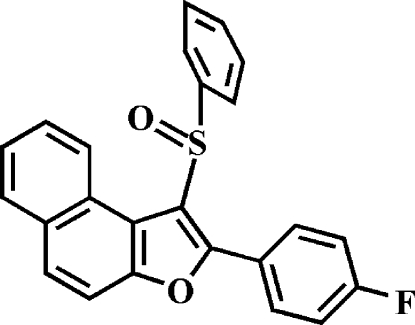


## Experimental

### 

#### Crystal data

C_24_H_15_FO_2_S
*M* *_r_* = 386.42
Triclinic, 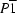

*a* = 7.2853 (2) Å
*b* = 10.1619 (3) Å
*c* = 12.3137 (4) Å
α = 103.439 (2)°
β = 90.486 (2)°
γ = 96.422 (2)°
*V* = 880.53 (5) Å^3^
*Z* = 2
Mo *K*α radiation
μ = 0.21 mm^−1^
*T* = 173 K
0.29 × 0.26 × 0.22 mm

#### Data collection

Bruker SMART APEXII CCD diffractometer
Absorption correction: multi-scan (*SADABS*; Bruker, 2009 ▶) *T*_min_ = 0.941, *T*_max_ = 0.954
16660 measured reflections
4392 independent reflections
3675 reflections with *I* > 2σ(*I*)
*R*_int_ = 0.037

#### Refinement

*R*[*F* ^2^ > 2σ(*F* ^2^)] = 0.040
*wR*(*F* ^2^) = 0.111
*S* = 1.05
4392 reflections
253 parameters
H-atom parameters constrained
Δρ_max_ = 0.38 e Å^−3^
Δρ_min_ = −0.33 e Å^−3^

### 

Data collection: *APEX2* (Bruker, 2009 ▶); cell refinement: *SAINT* (Bruker, 2009 ▶); data reduction: *SAINT*; program(s) used to solve structure: *SHELXS97* (Sheldrick, 2008 ▶); program(s) used to refine structure: *SHELXL97* (Sheldrick, 2008 ▶); molecular graphics: *ORTEP-3* (Farrugia, 1997 ▶) and *DIAMOND* (Brandenburg, 1998 ▶); software used to prepare material for publication: *SHELXL97*.

## Supplementary Material

Crystal structure: contains datablock(s) global, I. DOI: 10.1107/S1600536811034155/qm2024sup1.cif

Structure factors: contains datablock(s) I. DOI: 10.1107/S1600536811034155/qm2024Isup2.hkl

Supplementary material file. DOI: 10.1107/S1600536811034155/qm2024Isup3.cml

Additional supplementary materials:  crystallographic information; 3D view; checkCIF report

## Figures and Tables

**Table 1:** Hydrogen-bond geometry (Å, °)

*D*—H⋯*A*	*D*—H	H⋯*A*	*D*⋯*A*	*D*—H⋯*A*
C23—H23⋯O2^i^	0.95	2.53	3.277 (2)	136

Symmetry code: (i) 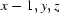.

**Fig. 1.**
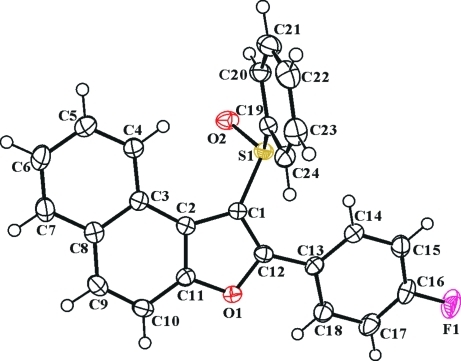
The molecular structure of the title compound with the atom numbering scheme. Displacement ellipsoids are drawn at the 50% probability level. H atoms are presented as small spheres of arbitrary radius.

## supplementary crystallographic information

### Comment

Many compounds containing a naphthofuran moiety have drawn much attention owing to their valuable biological properties (Goel & Dixit, 2004; Hagiwara *et al.*, 1999; Piloto *et al.*, 2005). As a part of our ongoing studies of the substituent effect on the solid state structures of 2–aryl–1–(phenylsulfinyl)naphtho[2,1–b]furan analogues (Choi *et al.*, 2009*a*,*b*), we report herein the crystal structure of the title compound.

In the title molecule (Fig. 1), the naphthofuran unit is essentially planar, with a mean deviation of 0.291 (1) Å from the least-squares plane defined by the thirteen constituent atoms. The 4–fluorophenyl ring makes the dihedral angle of 19.43 (4)° with the mean plane of the naphthofuran fragment. The diheral angle formed by the phenyl ring and the mean plane of the naphthofuran fragment is 85.83 (4)°. The crystal packing  is stabilized by weak intermolecular C—H···O hydrogen bonds between a phenyl H atom and the O atom of the sulfinyl group (Table 1; C23—H23···O2^i^).

### Experimental

77% 3-chloroperoxybenzoic acid (224 mg, 1.0 mmol) was added in small portions to a stirred solution of 2–(4–fluorophenyl)–1–(phenylsulfanyl)naphtho [2,1-b]furan (333 mg, 0.9 mmol) in dichloromethane (40 mL) at 273 K. After being stirred at room temperature for 4h, the mixture was washed with saturated sodium bicarbonate solution and the organic layer was separated, dried over magnesium sulfate, filtered andconcentrated at reduced pressure. The residue was purified by column chromatography (hexane-ethyl acetate, 2:1 v/v) to afford the title compound as a colorless solid [yield 65%, m.p. 473–474 K; Rf = 0.46 (hexane–ethyl acetate, 2:1 v/v)]. Single crystals suitable for X–ray diffraction were prepared by slow evaporation of a solution of the title compound in benzene at room temperature.

### Refinement

All H atoms were positioned geometrically and refined using a riding model, with C—H = 0.95 Å for aryl H atoms. *U*_iso_(H) = 1.2*U*_eq_(C) for aryl H atoms.

### Crystal data

**Table:**

C_24_H_15_FO_2_S	*Z* = 2
*M**_r_* = 386.42	*F*(000) = 400
Triclinic, *P*1	*D*_x_ = 1.457 Mg m^−^^3^
Hall symbol: -P 1	Mo *K*α radiation, λ = 0.71073 Å
*a* = 7.2853 (2) Å	Cell parameters from 5489 reflections
*b* = 10.1619 (3) Å	θ = 2.8–28.2°
*c* = 12.3137 (4) Å	µ = 0.21 mm^−^^1^
α = 103.439 (2)°	*T* = 173 K
β = 90.486 (2)°	Block, colourless
γ = 96.422 (2)°	0.29 × 0.26 × 0.22 mm
*V* = 880.53 (5) Å^3^	

### Data collection

**Table:**

Bruker SMART APEXII CCD diffractometer	4392 independent reflections
Radiation source: rotating anode	3675 reflections with *I* > 2σ(*I*)
graphite multilayer	*R*_int_ = 0.037
Detector resolution: 10.0 pixels mm^-1^	θ_max_ = 28.4°, θ_min_ = 1.7°
φ and ω scans	*h* = −9→9
Absorption correction: multi-scan (*SADABS*; Bruker, 2009)	*k* = −13→13
*T*_min_ = 0.941, *T*_max_ = 0.954	*l* = −16→16
16660 measured reflections	

### Refinement

**Table:**

Refinement on *F*^2^	Primary atom site location: structure-invariant direct methods
Least-squares matrix: full	Secondary atom site location: difference Fourier map
*R*[*F*^2^ > 2σ(*F*^2^)] = 0.040	Hydrogen site location: difference Fourier map
*wR*(*F*^2^) = 0.111	H-atom parameters constrained
*S* = 1.05	*w* = 1/[σ^2^(*F*_o_^2^) + (0.055*P*)^2^ + 0.2885*P*] where *P* = (*F*_o_^2^ + 2*F*_c_^2^)/3
4392 reflections	(Δ/σ)_max_ < 0.001
253 parameters	Δρ_max_ = 0.38 e Å^−^^3^
0 restraints	Δρ_min_ = −0.33 e Å^−^^3^

### Special details

**Table:**

Geometry. All esds (except the esd in the dihedral angle between two l.s. planes) are estimated using the full covariance matrix. The cell esds are taken into account individually in the estimation of esds in distances, angles and torsion angles; correlations between esds in cell parameters are only used when they are defined by crystal symmetry. An approximate (isotropic) treatment of cell esds is used for estimating esds involving l.s. planes.
Refinement. Refinement of F^2^ against ALL reflections. The weighted R-factor wR and goodness of fit S are based on F^2^, conventional R-factors R are based on F, with F set to zero for negative F^2^. The threshold expression of F^2^ > 2sigma(F^2^) is used only for calculating R-factors(gt) etc. and is not relevant to the choice of reflections for refinement. R-factors based on F^2^ are statistically about twice as large as those based on F, and R- factors based on ALL data will be even larger.

### Fractional atomic coordinates and isotropic or equivalent isotropic displacement parameters (Å^2^)

**Table:**

	*x*	*y*	*z*	*U*_iso_*/*U*_eq_	
S1	0.45928 (5)	0.63116 (4)	0.30349 (3)	0.02511 (11)	
O1	0.20787 (15)	0.47766 (10)	0.53486 (8)	0.0251 (2)	
O2	0.60815 (15)	0.55620 (12)	0.24620 (9)	0.0325 (3)	
F1	0.30192 (18)	1.08991 (10)	0.78434 (9)	0.0525 (3)	
C1	0.3299 (2)	0.52987 (14)	0.38112 (11)	0.0227 (3)	
C2	0.2802 (2)	0.38352 (14)	0.35730 (12)	0.0232 (3)	
C3	0.2860 (2)	0.27066 (14)	0.26339 (12)	0.0241 (3)	
C4	0.3429 (2)	0.28088 (15)	0.15649 (12)	0.0284 (3)	
H4	0.3837	0.3678	0.1435	0.034*	
C5	0.3403 (3)	0.16705 (17)	0.07079 (13)	0.0350 (4)	
H5	0.3778	0.1759	−0.0011	0.042*	
C6	0.2828 (3)	0.03789 (17)	0.08849 (14)	0.0396 (4)	
H6	0.2823	−0.0404	0.0287	0.047*	
C7	0.2276 (3)	0.02390 (16)	0.19071 (14)	0.0363 (4)	
H7	0.1896	−0.0645	0.2017	0.044*	
C8	0.2257 (2)	0.13876 (15)	0.28111 (13)	0.0277 (3)	
C9	0.1638 (2)	0.12265 (16)	0.38691 (13)	0.0307 (3)	
H9	0.1278	0.0334	0.3966	0.037*	
C10	0.1545 (2)	0.23082 (15)	0.47463 (13)	0.0285 (3)	
H10	0.1118	0.2198	0.5450	0.034*	
C11	0.2112 (2)	0.35935 (14)	0.45543 (12)	0.0244 (3)	
C13	0.2903 (2)	0.71658 (15)	0.56465 (11)	0.0246 (3)	
C14	0.3198 (2)	0.83664 (15)	0.52753 (13)	0.0292 (3)	
H14	0.3354	0.8320	0.4503	0.035*	
C15	0.3266 (2)	0.96241 (16)	0.60182 (14)	0.0327 (3)	
H15	0.3505	1.0441	0.5768	0.039*	
C16	0.2981 (2)	0.96647 (16)	0.71221 (14)	0.0346 (4)	
C17	0.2644 (3)	0.85122 (18)	0.75213 (13)	0.0376 (4)	
H17	0.2433	0.8575	0.8291	0.045*	
C18	0.2618 (2)	0.72569 (16)	0.67816 (12)	0.0315 (3)	
H18	0.2404	0.6449	0.7047	0.038*	
C19	0.2902 (2)	0.63310 (14)	0.19726 (12)	0.0243 (3)	
C12	0.2825 (2)	0.58147 (14)	0.48933 (12)	0.0238 (3)	
C20	0.3553 (2)	0.63478 (15)	0.09205 (12)	0.0284 (3)	
H20	0.4813	0.6249	0.0766	0.034*	
C21	0.2352 (2)	0.65096 (16)	0.00974 (13)	0.0341 (4)	
H21	0.2779	0.6505	−0.0630	0.041*	
C22	0.0532 (2)	0.66772 (17)	0.03334 (15)	0.0365 (4)	
H22	−0.0281	0.6813	−0.0228	0.044*	
C23	−0.0115 (2)	0.66481 (16)	0.13781 (15)	0.0352 (4)	
H23	−0.1373	0.6754	0.1531	0.042*	
C24	0.1062 (2)	0.64650 (15)	0.22047 (13)	0.0297 (3)	
H24	0.0616	0.6431	0.2922	0.036*	

### Atomic displacement parameters (Å^2^)

**Table:**

	*U*^11^	*U*^22^	*U*^33^	*U*^12^	*U*^13^	*U*^23^
S1	0.02566 (19)	0.02638 (19)	0.02358 (18)	0.00069 (14)	0.00323 (13)	0.00750 (13)
O1	0.0290 (5)	0.0258 (5)	0.0215 (5)	0.0044 (4)	0.0048 (4)	0.0068 (4)
O2	0.0266 (6)	0.0420 (6)	0.0325 (6)	0.0091 (5)	0.0074 (5)	0.0135 (5)
F1	0.0772 (8)	0.0330 (5)	0.0407 (6)	0.0154 (5)	−0.0018 (6)	−0.0089 (4)
C1	0.0234 (7)	0.0237 (6)	0.0214 (6)	0.0024 (5)	0.0024 (5)	0.0064 (5)
C2	0.0213 (6)	0.0250 (7)	0.0242 (7)	0.0029 (5)	0.0002 (5)	0.0074 (5)
C3	0.0223 (7)	0.0248 (7)	0.0252 (7)	0.0037 (5)	0.0000 (5)	0.0052 (5)
C4	0.0328 (8)	0.0270 (7)	0.0255 (7)	0.0038 (6)	0.0011 (6)	0.0062 (6)
C5	0.0454 (10)	0.0336 (8)	0.0247 (7)	0.0057 (7)	0.0028 (7)	0.0039 (6)
C6	0.0537 (11)	0.0273 (8)	0.0322 (8)	0.0022 (8)	−0.0007 (8)	−0.0027 (6)
C7	0.0447 (10)	0.0239 (7)	0.0377 (9)	0.0003 (7)	−0.0002 (7)	0.0040 (6)
C8	0.0266 (7)	0.0262 (7)	0.0300 (7)	0.0030 (6)	−0.0008 (6)	0.0064 (6)
C9	0.0310 (8)	0.0261 (7)	0.0367 (8)	0.0004 (6)	0.0023 (7)	0.0122 (6)
C10	0.0279 (7)	0.0313 (7)	0.0293 (7)	0.0037 (6)	0.0054 (6)	0.0131 (6)
C11	0.0236 (7)	0.0257 (7)	0.0240 (7)	0.0045 (6)	0.0023 (5)	0.0054 (5)
C13	0.0226 (7)	0.0275 (7)	0.0232 (7)	0.0057 (6)	0.0011 (5)	0.0039 (5)
C14	0.0318 (8)	0.0289 (7)	0.0265 (7)	0.0045 (6)	0.0044 (6)	0.0048 (6)
C15	0.0331 (8)	0.0261 (7)	0.0378 (8)	0.0038 (6)	0.0021 (7)	0.0048 (6)
C16	0.0377 (9)	0.0287 (8)	0.0328 (8)	0.0102 (7)	−0.0034 (7)	−0.0051 (6)
C17	0.0506 (10)	0.0412 (9)	0.0218 (7)	0.0167 (8)	0.0005 (7)	0.0036 (6)
C18	0.0396 (9)	0.0322 (8)	0.0243 (7)	0.0109 (7)	0.0002 (6)	0.0069 (6)
C19	0.0273 (7)	0.0208 (6)	0.0254 (7)	0.0032 (5)	0.0015 (6)	0.0064 (5)
C12	0.0228 (7)	0.0265 (7)	0.0233 (6)	0.0033 (6)	0.0009 (5)	0.0084 (5)
C20	0.0282 (7)	0.0304 (7)	0.0281 (7)	0.0041 (6)	0.0045 (6)	0.0096 (6)
C21	0.0400 (9)	0.0350 (8)	0.0292 (8)	0.0010 (7)	0.0007 (7)	0.0129 (6)
C22	0.0361 (9)	0.0332 (8)	0.0409 (9)	0.0022 (7)	−0.0092 (7)	0.0112 (7)
C23	0.0258 (8)	0.0317 (8)	0.0475 (9)	0.0041 (6)	0.0004 (7)	0.0082 (7)
C24	0.0299 (8)	0.0270 (7)	0.0319 (8)	0.0044 (6)	0.0062 (6)	0.0058 (6)

### Geometric parameters (Å, °)

**Table:**

S1—O2	1.4840 (11)	C10—H10	0.9500
S1—C1	1.7655 (15)	C13—C14	1.395 (2)
S1—C19	1.7939 (15)	C13—C18	1.398 (2)
O1—C11	1.3650 (16)	C13—C12	1.4623 (19)
O1—C12	1.3690 (17)	C14—C15	1.384 (2)
F1—C16	1.3554 (17)	C14—H14	0.9500
C1—C12	1.3761 (19)	C15—C16	1.369 (2)
C1—C2	1.4505 (19)	C15—H15	0.9500
C2—C11	1.376 (2)	C16—C17	1.371 (2)
C2—C3	1.4316 (19)	C17—C18	1.383 (2)
C3—C4	1.407 (2)	C17—H17	0.9500
C3—C8	1.429 (2)	C18—H18	0.9500
C4—C5	1.371 (2)	C19—C20	1.387 (2)
C4—H4	0.9500	C19—C24	1.388 (2)
C5—C6	1.399 (2)	C20—C21	1.384 (2)
C5—H5	0.9500	C20—H20	0.9500
C6—C7	1.358 (2)	C21—C22	1.380 (2)
C6—H6	0.9500	C21—H21	0.9500
C7—C8	1.415 (2)	C22—C23	1.379 (3)
C7—H7	0.9500	C22—H22	0.9500
C8—C9	1.421 (2)	C23—C24	1.384 (2)
C9—C10	1.359 (2)	C23—H23	0.9500
C9—H9	0.9500	C24—H24	0.9500
C10—C11	1.399 (2)		
			
O2—S1—C1	109.66 (7)	C14—C13—C12	122.77 (13)
O2—S1—C19	106.56 (7)	C18—C13—C12	118.58 (14)
C1—S1—C19	100.32 (7)	C15—C14—C13	120.83 (14)
C11—O1—C12	107.08 (11)	C15—C14—H14	119.6
C12—C1—C2	106.96 (12)	C13—C14—H14	119.6
C12—C1—S1	122.10 (11)	C16—C15—C14	118.52 (15)
C2—C1—S1	130.23 (11)	C16—C15—H15	120.7
C11—C2—C3	118.81 (13)	C14—C15—H15	120.7
C11—C2—C1	104.59 (12)	F1—C16—C15	118.28 (15)
C3—C2—C1	136.60 (13)	F1—C16—C17	119.01 (15)
C4—C3—C8	118.60 (13)	C15—C16—C17	122.71 (14)
C4—C3—C2	124.99 (13)	C16—C17—C18	118.67 (15)
C8—C3—C2	116.40 (13)	C16—C17—H17	120.7
C5—C4—C3	120.89 (15)	C18—C17—H17	120.7
C5—C4—H4	119.6	C17—C18—C13	120.61 (15)
C3—C4—H4	119.6	C17—C18—H18	119.7
C4—C5—C6	120.45 (15)	C13—C18—H18	119.7
C4—C5—H5	119.8	C20—C19—C24	120.76 (14)
C6—C5—H5	119.8	C20—C19—S1	116.63 (12)
C7—C6—C5	120.35 (15)	C24—C19—S1	122.28 (11)
C7—C6—H6	119.8	O1—C12—C1	109.83 (12)
C5—C6—H6	119.8	O1—C12—C13	114.08 (12)
C6—C7—C8	121.09 (15)	C1—C12—C13	136.09 (14)
C6—C7—H7	119.5	C21—C20—C19	119.39 (15)
C8—C7—H7	119.5	C21—C20—H20	120.3
C7—C8—C9	120.40 (14)	C19—C20—H20	120.3
C7—C8—C3	118.61 (14)	C22—C21—C20	120.00 (15)
C9—C8—C3	120.99 (13)	C22—C21—H21	120.0
C10—C9—C8	122.07 (14)	C20—C21—H21	120.0
C10—C9—H9	119.0	C23—C22—C21	120.45 (15)
C8—C9—H9	119.0	C23—C22—H22	119.8
C9—C10—C11	116.22 (14)	C21—C22—H22	119.8
C9—C10—H10	121.9	C22—C23—C24	120.23 (15)
C11—C10—H10	121.9	C22—C23—H23	119.9
O1—C11—C2	111.49 (12)	C24—C23—H23	119.9
O1—C11—C10	123.09 (13)	C23—C24—C19	119.14 (15)
C2—C11—C10	125.40 (13)	C23—C24—H24	120.4
C14—C13—C18	118.63 (13)	C19—C24—H24	120.4
			
O2—S1—C1—C12	134.17 (12)	C9—C10—C11—C2	−2.3 (2)
C19—S1—C1—C12	−113.94 (13)	C18—C13—C14—C15	2.0 (2)
O2—S1—C1—C2	−34.89 (15)	C12—C13—C14—C15	−179.88 (14)
C19—S1—C1—C2	77.00 (14)	C13—C14—C15—C16	−2.0 (2)
C12—C1—C2—C11	−1.64 (16)	C14—C15—C16—F1	−178.94 (15)
S1—C1—C2—C11	168.69 (12)	C14—C15—C16—C17	0.4 (3)
C12—C1—C2—C3	178.01 (16)	F1—C16—C17—C18	−179.64 (15)
S1—C1—C2—C3	−11.7 (3)	C15—C16—C17—C18	1.0 (3)
C11—C2—C3—C4	176.20 (14)	C16—C17—C18—C13	−0.9 (3)
C1—C2—C3—C4	−3.4 (3)	C14—C13—C18—C17	−0.6 (2)
C11—C2—C3—C8	−2.6 (2)	C12—C13—C18—C17	−178.74 (15)
C1—C2—C3—C8	177.77 (15)	O2—S1—C19—C20	−31.41 (13)
C8—C3—C4—C5	0.2 (2)	C1—S1—C19—C20	−145.68 (12)
C2—C3—C4—C5	−178.65 (15)	O2—S1—C19—C24	155.11 (12)
C3—C4—C5—C6	−0.7 (3)	C1—S1—C19—C24	40.84 (13)
C4—C5—C6—C7	0.5 (3)	C11—O1—C12—C1	1.02 (16)
C5—C6—C7—C8	0.4 (3)	C11—O1—C12—C13	−179.62 (12)
C6—C7—C8—C9	178.66 (16)	C2—C1—C12—O1	0.40 (16)
C6—C7—C8—C3	−1.0 (3)	S1—C1—C12—O1	−170.89 (10)
C4—C3—C8—C7	0.7 (2)	C2—C1—C12—C13	−178.75 (16)
C2—C3—C8—C7	179.59 (14)	S1—C1—C12—C13	10.0 (2)
C4—C3—C8—C9	−178.94 (14)	C14—C13—C12—O1	−164.53 (14)
C2—C3—C8—C9	0.0 (2)	C18—C13—C12—O1	13.57 (19)
C7—C8—C9—C10	−177.87 (16)	C14—C13—C12—C1	14.6 (3)
C3—C8—C9—C10	1.7 (2)	C18—C13—C12—C1	−167.30 (17)
C8—C9—C10—C11	−0.7 (2)	C24—C19—C20—C21	0.4 (2)
C12—O1—C11—C2	−2.16 (16)	S1—C19—C20—C21	−173.16 (12)
C12—O1—C11—C10	176.51 (14)	C19—C20—C21—C22	1.2 (2)
C3—C2—C11—O1	−177.37 (12)	C20—C21—C22—C23	−1.8 (2)
C1—C2—C11—O1	2.34 (16)	C21—C22—C23—C24	0.7 (3)
C3—C2—C11—C10	4.0 (2)	C22—C23—C24—C19	0.9 (2)
C1—C2—C11—C10	−176.29 (14)	C20—C19—C24—C23	−1.5 (2)
C9—C10—C11—O1	179.24 (13)	S1—C19—C24—C23	171.74 (12)

### Hydrogen-bond geometry (Å, °)

**Table:**

*D*—H···*A*	*D*—H	H···*A*	*D*···*A*	*D*—H···*A*
C23—H23···O2^i^	0.95	2.53	3.277 (2)	136.

Symmetry codes: (i) *x*−1, *y*, *z*.
